# Circadian Alterations Increase with Progression in a Patient-Derived Cell Culture Model of Breast Cancer

**DOI:** 10.3390/clockssleep3040042

**Published:** 2021-11-12

**Authors:** Hui-Hsien Lin, Stephanie R. Taylor, Michelle E. Farkas

**Affiliations:** 1 Department of Chemistry, University of Massachusetts Amherst, Amherst, MA 01003, USA; hlin@umass.edu; 2 Department of Computer Science, Colby College, Waterville, ME 04901, USA

**Keywords:** breast cancer, cancer progression, circadian rhythm, metastatic cells, rhythmicity

## Abstract

Circadian rhythm disruption can elicit the development of various diseases, including breast cancer. While studies have used cell lines to study correlations between altered circadian rhythms and cancer, these models have different genetic backgrounds and do not mirror the changes that occur with disease development. Isogenic cell models can recapitulate changes across cancer progression. Hence, in this study, a patient-derived breast cancer model, the 21T series, was used to evaluate changes to circadian oscillations of core clock protein transcription as cells progress from normal to malignant states. Three cell lines were used: H16N2 (normal breast epithelium), 21PT (atypical ductal hyperplasia), and 21MT-1 (invasive metastatic carcinoma). The cancerous cells are both HER2+. We assessed the transcriptional profiles of two core clock proteins, BMAL1 and PER2, which represent a positive and negative component of the molecular oscillator. In the normal H16N2 cells, both genes possessed rhythmic mRNA oscillations with close to standard periods and phases. However, in the cancerous cells, consistent changes were observed: both genes had periods that deviated farther from normal and did not have an anti-phase relationship. In the future, mechanistic studies should be undertaken to determine the oncogenic changes responsible for the circadian alterations found.

## 1. Introduction

The circadian clock is a hierarchical timing system that regulates physiological, behavioral, and metabolic functions across a 24 h day–night cycle and maintains temporal tissue homeostasis in coordination with the external environment [1,2]. Peripheral clocks are entrained by a central core clock located in the suprachiasmatic nucleus (SCN) and are necessary for normal tissue functioning, including cell development [3,4]. At the molecular level, the core clock consists of a well-characterized transcriptional–translational feedback loop (TTFL), where CLOCK and BMAL1 bind to an E-box promoter to drive the expression of other clock genes, including *CRY* and *PER*. Subsequently, CRY and PER accumulate in the cytoplasm during the day to form complexes, which translocate back to the nucleus to suppress the activity of CLOCK and BMAL1 [2]. Disrupted circadian rhythms have been associated with different types of cancer, including but not limited to breast [5,6,7,8], colon [9], and prostate cancers [10]. For breast cancer, in particular, epidemiological studies have shown that long-term night shift workers have a higher risk of developing the disease [11,12,13]. Furthermore, both in vitro and in vivo studies have indicated that mutation or dysregulation of clock genes can lead to the initiation of breast tumor growth and metastases [5,6,7,8].

Among women in the United States, breast cancer is the most commonly diagnosed cancer type, with the second highest cancer death rate following lung and bronchus cancer [14]. Female breast tissue is subject to more frequent remodeling than other types [15]. For example, during pregnancy, lactation, and involution, the mammary epithelium undergoes multiple cycles of proliferation and cell death [16,17]. As a result, the mammary gland is more prone to abnormalities, which can result in cancer [18]. While it has been shown that genetic mutations and epigenetic modifications contribute to breast cancer progression and development [19,20], the molecular mechanisms of such processes are still unclear.

Various breast cancer cell lines (e.g., MCF-7, MDA-MB-231, SKBR3, and many others) are frequently used for studying the disease and potential therapeutics for treating it [21,22,23]. Although these cellular models are generally categorized into different breast cancer subtypes [24], they each represent only a single stage of a progressive disease. As a complementary approach, a series of cell lines representing disease progression can be used. There are a few such models that have been used to study breast cancer development, including the MCF10 human isogenic series [25,26,27] and the 4T1 syngeneic mouse model [28,29]. While each represents different stages of cancer and can be related to human disease, the MCF10 series was derived via laboratory-based genetic manipulations (including an H-Ras mutation rarely found in breast cancer patients), and the 4T1 series originated from a single, spontaneously arising mammary tumor in a BALB/c mouse. Neither may truly reflect the natural evolution of human breast cancer.

Among progressive cancer models, the 21T series of cells is particularly relevant, since each cell type was isolated from the same patient, who originally had infiltrating and intraductal carcinoma and later developed metastases to the lung [30]. Four cell lines comprise the model: H16N2, tumor-adjacent non-cancerous breast cells; 21PT, Atypical Ductal Hyperplasia (ADH) cells, which are non-tumorigenic and non-metastatic; 21NT, Ductal Carcinoma In Situ (DCIS), which are tumorigenic and non-metastatic; and 21MT, Invasive Metastatic Carcinoma (IMC), which are both tumorigenic and metastatic. Two distinct populations exist within the 21MT cell line, which was further separated into 21MT-1 and 21MT-2. 21MT-1 are highly heterogeneous, tend to grow in clusters, and do not form confluent monolayers, while 21MT-2 are homogeneous polygonal cells that grow as monolayers [30]. All cancerous cells of the 21T series overexpress *ERBB2* compared to normal H16N2 cells [30]. *ERBB2* encodes the human epidermal growth factor receptor 2 (HER2), which is overexpressed, amplified, or both in several human malignancies including breast, ovarian, and colon cancers [31]. Overexpression of HER2 in human tumor cells is closely associated with increased angiogenesis, higher rates of cell survival, and poor clinical outcomes [32].

Disruption of circadian rhythms has been shown to trigger cancer development, and vice versa, malignant transformations have led to disturbances of circadian clocks [5,6,7,8,13]. In one of our previous studies, we showed that circadian clocks are disrupted across the MCF10 series of cells representing human breast cancer [33]. We found that from benign to metastatic states, *PER2* exhibited relatively stable oscillations compared to *BMAL1*, whose periods were altered over a wide circadian range. As mentioned above, while the MCF10 series represents progressive stages of breast cancer, it originated with an H-Ras mutation, not commonly found in patients with the disease. To evaluate circadian changes in a more relevant model, we used the human 21T series of cells in the present study. This work represents the first assessment of circadian oscillations in a patient-derived model of breast cancer.

We used three cell lines from the 21T series, H16N2, 21PT, and 21MT-1, representing disease progression from benign to metastatic, to determine changes in the expression profiles of core circadian clock genes. We focused on a positive and negative component of the molecular clock oscillator, *BMAL1* and *PER2*, respectively. The mRNA expressions of *BMAL1* and *PER2* were tracked in a time-dependent manner via RT-PCR assessments. Our transcriptional data indicate that the non-oncogenic H16N2 cells possess robust circadian patterns, while 21PT and 21MT-1 have consistently less reliable rhythms, with altered characteristics. These results support our hypothesis that circadian rhythms deviate from normal when cells undergo malignant transformations. In further studies, a luciferase or fluorescent reporter should be used to track the promoter activity of clock genes and/or protein expression to generate high-resolution data for a complete assessment of circadian parameters in this model.

## 2. Results

In the current study, we sought to evaluate circadian alterations in a progressive, patient-derived breast cancer model to ascertain whether changes occurred with disease status. The 21T series of cell lines is considered a unique experimental model of breast cancer progression, since there are no other models that originated from a single patient. We used three cell lines from the human breast cancer 21T series, H16N2, 21PT, and 21MT-1, which were isolated from a single patient and have the same genetic background but different characteristics. H16N2 is derived from the normal epithelia, 21PT is derived from atypical ductal hyperplasia, and 21MT-1 is derived from the pleural effusion of lung metastasis [30]. This is an excellent model to recapitulate the de novo processes arising during breast cancer evolution.

**Figure 1:**
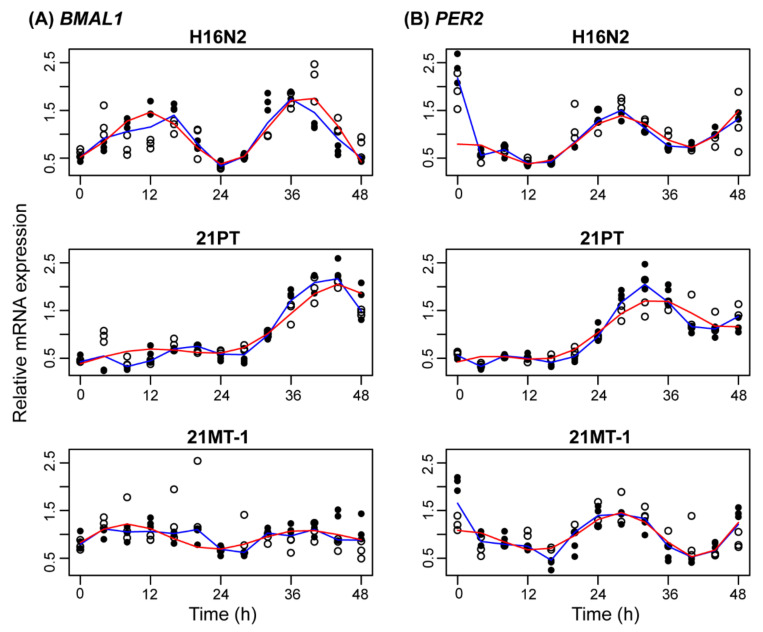
Relative mRNA expression of (**A**) *BMAL1* and (**B**) *PER2* across the 21T series of cells. Shown are the mRNA expression levels relative to the mean over time for each biological replicate (sample number (n) was 6 for all but 4 time points in 21MT-1, where n = 5). The experiment was conducted twice (n = 3 for each); open and closed circles are used to differentiate between the two. The median and best-fit damped cosine curves are shown in blue and red, respectively. The coefficient of determination (R^2^) indicates a lack of fit for *BMAL1* 21MT-1 (R^2^ = −0.01), low-quality fits for *PER2* in H16N2 (R^2^ = 0.27) and *PER2* in 21MT-1 (R^2^ = 0.46), and medium-quality fits for the remaining curves (R^2^ = 0.58 for *BMAL1* in H16N2, 0.84 for *PER2* in 21PT, and 0.86 for *BMAL1* in 21MT-1).

We focused on the expression of two core circadian clock components, one from the positive and the other the negative arm of the feedback loop, *BMAL1* and *PER2*, respectively. mRNA expression of both was evaluated for each of the three 21T cell lines, using RT-PCR (Figure 1), with sampling once every four hours from three biological replicates per cell line, over the course of two cycles from two separate experiments. We sought to determine whether each time series was rhythmic and, if so, its circadian properties (such as peak time; Table 1). To assess rhythmicity, we chose multiple, complementary methods that allowed for replicates and were flexible with respect to waveform and missing data: RAIN (arbitrary waveform) [34], a Lomb–Scargle permutation test (allows for missing data) [35], JTK-Cycle (compares reference curves to data) [36], and ECHO (fits data to a sine curve with amplitude changes over time) [37]. To assess circadian properties and the quality of rhythmicity, we fit each time series (with replicates) to a damped cosine curve.

All tests for rhythmicity indicated that the non-oncogenic H16N2 and non-tumorigenic, non-metastatic 21PT cells are rhythmic, where *p* < 0.001 for each. The patterns for the *BMAL1* and *PER2* transcripts from 21MT-1 cells were found to be rhythmic using most tests (*p* < 0.001), with the exception of the Lomb–Scargle permutation test for *BMAL1*, where *p* = 0.08. Given that each data set was determined to be rhythmic by more than one test, we sought to further assess the characteristics of the oscillation patterns.

The normal breast epithelial H16N2 cells showed *BMAL1* peaks at ~12–16 h and 32–36 h, while those for *PER2* were at ~0–4 h and ~24–28 h. Hence, as expected for these positive and negative elements of the core clock, the phase difference between the two is approximately 12 h, with oscillation patterns in opposition to each other. A slowly amplifying cosine curve with an increasing baseline fit the data relatively well, with the exception of the first time point for *PER2* (R^2^ = 0.58 for *BMAL1* and 0.27 for *PER2*). Over 48 h, the baseline of relative expression increased by 0.16 for *BMAL1* and 0.86 for PER2, and the oscillations grew exponentially with rates of 0.02/h for *BMAL1* and 0.01/h for *PER2* (Table 1, Tables S1 and S2). It also estimated peak times, where *BMAL1* peaked 11.4 h and *PER2* peaked 0.5 h into each cycle. The periods of both *BMAL1* and *PER2* were determined to be 26.5 h with a small estimation error (*BMAL1* 95% CI: 25–27.7 h; *PER2* 95% CI: 25–28.2 h).

The 21PT (non-tumorigenic and non-metastatic) cells showed oscillations with increasing amplitude. Visual inspection shows different phases from H16N2 with *BMAL1* peaking at ~16–20 h and 40–44 h, while *PER2* peaks at ~8–12 h and 28–32 h. We confirmed the rhythmic patterns were different with robustDODR applied to the time series after removing a linear trend (*p* < 0.001) [38], comparing each reporter across cell lines. Unlike in the normal cells, the transcriptional patterns here were not anti-phase but still had consistent changes (an approximately 8 h offset) for both *BMAL1* and *PER2* in all samples. Curve fitting also yielded reasonable fits for this cell line (R^2^ = 0.86 for *BMAL1* and 0.84 for *PER2*; Table 1, Tables S3 and S4). Compared to H16N2 cells, 21PT oscillations showed larger growth rates (0.05/h for *BMAL1* and 0.03/h for *PER2*) and steeper baselines (relative expression of *BMAL1* increased by 1.09 over 48 h and *PER2* by 1.54). The periods were also both longer than those of H16N2 transcripts, and there was more uncertainty in their estimates. The 95% confidence interval for the period of *BMAL1* was 32–42 h for *PER2* was 28.4–34.3 h.

The 21MT-1 (tumorigenic, metastatic) cells showed oscillations with the least circadian characteristics. Compared to H16N2, the transcriptional patterns for both *BMAL1* and *PER2* were statistically different (robustDODR applied to linearly de-trended time series was *p* < 0.001). For *BMAL1*, curve fitting failed to explain the variance in the data (R^2^ = −0.01), so we do not report circadian parameters. For *PER2*, the time series for 21MT-1 shows less reliable estimates of parameters and is less sinusoidal (R^2^ = 0.46). Confidence intervals for the parameter estimates of *PER2* are wide (95% CI for period: 25–29.4 h; 95% CI for growth rate: 0.008–0.04; Table S5). As in the 21PT cells, there were only 8 h between the peaks of *BMAL1* (8 h into each cycle) and *PER2* (0 h into each cycle).

Additionally, to assess possible outcomes of mRNA expression changes at the protein level, we conducted time-course Western blot analyses for BMAL1 and PER2 with the three members of the 21T series of cells evaluated above (Figure S1). We fit each of the time series to damped cosine curves (Figure S2). Unlike for mRNA expression patterns, the sinusoidal curves could not be adequately fit to the time series, resulting in low goodness-of-fit measures. BMAL1 and PER2 were not observed to have a consistent phase relationship. While some of the statistical tests for rhythmicity identified particular time series as rhythmic (Tables S6 and S7), these results were too inconsistent to be considered reliable, as confirmed by visual inspection. This outcome is perhaps due to the nature of the technique, discussed further below.

## 3. Discussion

Various experimental platforms have been used to investigate the correlation between altered circadian rhythms and cancer development. These include murine models with spontaneously developing or implanted tumors [39,40], individual cancer cell lines representing specific subtypes (e.g., MDA-MB-231 or MCF7, as mentioned above) [8,41,42], and series of cells representing cancer progression (e.g., 4T1 or MCF10 series, as mentioned above) [33,43]. Studies using these models typically show that the expression of clock genes and/or proteins is disrupted (i.e., with arrhythmic oscillation patterns or diminished expression levels) via RT-PCR or Western blotting assessments. However, the data obtained do not reflect progressive oncogenic changes, since non-related cell lines have diverse genetic backgrounds, and the other models were not derived from patients carrying breast cancer. At the same time, assessments in clinical studies often involve only a single time point [6,7,44,45]. However, the nature of circadian rhythms is dynamic, and it is important to carry out direct comparisons as a function of time. We recognize the difficulty in obtaining/using patient samples in such a manner, and so, in the present study, we used primary and metastatic tumor cells along with those of the normal breast epithelium, derived from the same individual, to directly compare circadian properties in a time-dependent manner.

The foundation of circadian rhythmicity is the oscillation of molecular clocks for a period of approximately 24 h [46,47]. During 24 h cycling, the peak times of positive and negative core clock regulators (i.e., *BMAL1* and *PER2*, respectively) exhibit a nearly 12 h delay from one another, resulting in an anti-phase relationship [46]. While this is the case in healthy cells, in disease states, various alterations (e.g., changes to period [47], phase [48], and amplitude [49]) may occur. As shown in Figure 1 and Table 1, *BMAL1* and *PER2* transcripts from normal breast epithelial H16N2 cells showed rhythmic, anti-phase mRNA oscillations (~12 h differences in peak times) with similar periods that were close to 24 h (estimates were 26.5 h for both) and good fits. However, longer and dissimilar periods (32.0 h for *BMAL1* and 30.5 h for *PER2* in 21PT; 30.5 h for *BMAL1* and 27.1 h for *PER2* in 21MT-1), and altered phase relationships (~8 h shifts in peak times between *BMAL1* and *PER2*) were found in the cancerous 21PT (ADH) and 21MT-1 (IMC) cells.

Few studies have analyzed changes in circadian characteristics using progressive cancer models, and this is the first such study in a disease-relevant model of breast cancer. Nonetheless, we compared our findings to those of others and strikingly observed similarities. For example, Relógio et al., discovered that metastatic human skin and colorectal cancer cell lines tended to have longer periods and altered phases compared to the respective normal epithelial cells [47,50]. Deviations from period and phase are also seen in our data, where 21PT cells exhibited consistent changes (i.e., ~8 h phase offset with similar growth rates) for *BMAL1* and *PER2*, while 21MT-1 showed contrasting alterations for the two genes (i.e., ~8 h phase offset with two opposing growth trends, one increasing and the other decreasing) and the worst sinusoidal curve fits overall. It is surprising to us that different cancers can have similar changes in circadian characteristics. Broadly, our results support the hypothesis that circadian rhythms are increasingly disrupted in breast cancer.

Although it is unclear how circadian changes occur in the cancerous 21PT and 21MT-1 cells, molecular profiling of the cells may provide some clues. Several studies have characterized the genetic profiles of cells from the 21T series [30,51,52,53,54]. Band et al., showed that compared to normal epithelial H16N2 cells, the others were *HER2* positive (overexpressing *ERBB2*) [30]. It is known that *ERBB2* is closely associated with *NR1D1* [55], the gene that encodes REV-ERBα (a clock protein involved in the secondary clock TTFL), and high levels of *NR1D1* have frequently been found in breast cancer patients [56]. In addition to being *HER2* positive, 21MT-1 cells had hyper-activated p-Ser^473^ Akt [52], a kinase involved in the PI3K/Akt/mTOR pathway frequently activated in breast cancer [57,58]. AKT phosphorylates CLOCK and BMAL1 and inhibits their nuclear localization [59,60], and the mTOR pathway has been shown to regulate circadian entrainment in the SCN [61]. Taken together, abnormal *ERBB2*/*NR1D1* expression and activated Akt/mTOR pathways may all contribute to the prolonged periods and altered phases of *BMAL1* and *PER2* in the cancerous 21PT and 21MT-1 cells. Both aspects should be examined further to determine their potential contributions to altered circadian oscillations.

To evaluate whether changes in transcriptional expression can be detected at the translational level, we performed time-course Western blot analyses of BMAL1 and PER2 in the three cell lines from the 21T series. Our results showed that there was no clear and consistent phase relationship between BMAL1 and PER2 in any of them (Figures S1 and S2). Although some statistical analyses revealed rhythmicity in certain expression patterns (Tables S6 and S7), it is likely that the changes were too small to be reliable. Several studies have investigated temporal changes in clock protein expression using different disease models; however, while some showed clear and detectable protein oscillations [62,63], others did not, perhaps due to the low resolution of Western blotting [64,65]. We also noted subtle changes in GAPDH expression over time; however, the minor instability of a reference protein has been shown previously in various cell models [50,66,67].

The poor correlation between observed mRNA and protein expression could also be induced by changes to transcriptional regulation, post-transcriptional modifications, protein half-lives, and technical errors and noise in either or both mRNA and protein experiments [68,69,70]. As an example, Robles et al., showed that post-transcriptional modifications caused a phase delay of 6 h between cycling transcripts and corresponding proteins [69]. Future studies with this system should use luciferase reporters to acquire high-resolution data at both the promoter and translational levels, which will enable more detailed visualizations of oscillations and thorough analyses.

## 4. Materials and Methods

### 4.1. Cell Culture

H16N2, 21PT, and 21MT-1 cell lines were obtained from Prof. D. Joseph Jerry (Veterinary and Animal Sciences, UMass Amherst, MA, USA), who received them directly from Dr. Vimla Band, who originally isolated the cells. Cells were maintained in MEM (Gibco, Waltham, MA, USA), with 1% penicillin–streptomycin (Gibco), 1 mM HEPES (Hyclone, Logan, UT, USA), 1 mM sodium pyruvate (Gibco), 2 mM L-glutamine (Gibco), 10% fetal bovine serum (FBS; Corning, Corning, NY, USA), 15 μg/mL gentamicin (Fisher Scientific, Waltham, MA, USA), 1 μg/mL insulin (Sigma, St. Louis, MO, USA), 12.5 ng/mL epidermal growth factor (EGF; Gibco), and 1 μg/mL hydrocortisone (Sigma). All cells were incubated at 37 °C under 5% CO_2_.

### 4.2. Synchronization of Cells by Serum Shock

A 2 mL volume of cells at a density of 2 × 10^5^ cells/mL was plated in 35 mm culture dishes and incubated for approximately 24 h to reach 100% confluence. Culture media were discarded, and cells were washed once with 2 mL of phosphate-buffered saline (PBS; Gibco). Cells were then starved in MEM medium without any supplements for 12 h. After starvation, cells were serum shocked using growth medium containing 50% FBS for 2 h, followed by a wash with PBS, and returned to starvation conditions (culture in MEM medium without any supplements).

### 4.3. RNA Extraction and cDNA Synthesis

Cells were collected at the first time point immediately following serum shock (T = 0) and every 4 h thereafter for 48 h. Total RNA was extracted via TRIzol Reagent (Gibco) according to the manufacturer’s instructions. Briefly, 1 mL of TRIzol was added to lyse the cells, and cell lysates were transferred to microcentrifuge tubes. Cell lysates were incubated at rt for 5 min to allow complete dissociation of nucleoprotein complexes. After the addition of 200 μL chloroform per 1 mL of TRIzol, samples were shaken vigorously by hand for 15 s and incubated at rt for 3 min. Samples were then centrifuged at 12,000× *g* for 15 min at 4 °C to separate the RNA-containing, upper aqueous phase, from the lower chloroform phase. RNA samples were further purified via the PureLink RNA kit (Ambion, Austin, TX, USA) according to the manufacturer’s instructions. Total RNA concentration was determined by Nanodrop UV/Vis (Thermo Fisher Scientific, Waltham, MA, USA). A 1 μg amount of total RNA was reverse-transcribed to cDNA using 50 μM random hexamers, 40 U/μL RNaseOut, 10 mM dNTPs, and 200 U/μL SuperScript IV Reverse Transcriptase (Thermo Fisher Scientific).

### 4.4. Real-Time PCR (RT-PCR)

RT-PCR was performed in 96-well plates. Each reaction (20 μL per well) consisted of 100 ng cDNA, 10 μL iTaq universal SYBR Green Supermix (Bio-Rad, Hercules, CA, USA), 4 μM of each respective forward and reverse primer, and RNAse free water (Fisher) to a final volume of 20 μL. All DNA primers were purchased from Integrated DNA Technologies (Coralville, IA, USA). The following sequences were used: *GAPDH* forward (5′-CTT CTT TTG CGT CGC CAG CC-3′), reverse (5′-ATT CCG TTG ACT CCG ACC TTC-3′); *BMAL1* forward (5′-CTA CGC TAG AGG GCT TCC TG-3′), reverse (5′-CTT TTC AGG CGG TCA GCT TC-3′); *PER2* forward (5′-TGT CCC AGG TGG AGA GTG GT-3′), reverse (5′-TGT CAC CGC AGT TCA AAC GAG-3′). After brief centrifugation, samples were analyzed via the CFX Connect real-time system (Bio-Rad) programmed with an initial activation at 95 °C for 3 min, followed by 40 cycles of 95 °C denaturation for 10 s, and 60 °C annealing/extension for 30 s. Relative *BMAL1* and *PER2* expression levels were determined by comparing the *C_t_* values of *BMAL1* and *PER2* to *GAPDH* controls via the 2^∧^ΔΔC_t_ method [71]. Three biological replicates and three technical replicates per biological replicate were analyzed for each condition.

### 4.5. Rhythmicity Tests

Rhythmicity tests were performed using R packages (www.r-project.org). RAIN (rain v1.14.0) was used for a period of 24 h. The Lomb–Scargle permutation test (randlsp from lomb package v1.2; using the peak of the periodogram in the range of 6 to 50 h) used 5000 random permutations [35]. Metacycle v1.2.0 [72] was used to run the JTK-Cycle test. ECHO (echo.find v3.0) was used to test for periodicity in the range of 24 to 32 h, after linearly de-trending [37]. Following the tests for rhythmicity, a test for differential rhythmicity was used for each pair of cell lines with the DODR R package (v.0.99.2) [38].

### 4.6. Curve Fitting

Each mRNA time series (with 5–6 biological replicates) was fit to a “damped” cosine curve with a linear baseline: $Ae^{-\lambda t}\cos\left(2\pi \frac{t-\theta }{\tau }\right)+mt+b$, where *A* is amplitude, *λ* is damping rate, *θ* is phase, *τ* is intrinsic period, *m* is baseline slope, and *b* is *y*-intercept. *λ* was allowed to vary between −0.05 (a growing curve) and 0.05 (a damping curve). As in ECHO, each point was weighted by the inverse of the variance at its time step. The R function nls was used to estimate the coefficients and compute the 95% confidence intervals.

## 5. Conclusions

In this study, we report the first findings of circadian disruption in a progressive series of human, breast cancer patient-derived cells. We evaluated the oscillations of mRNA expression for two core clock components, *BMAL1* and *PER2*, in a time-dependent manner, in cell lines across the 21T series. Normal epithelial H16N2 cells displayed standard oscillation patterns. The two cancerous cell types, 21PT and 21MT-1, exhibited periods that deviated farther from 24 h and were less consistent between the *BMAL1* and *PER2* transcripts, which were also not anti-phase with the expected 12 h differences in peak times.

Our analyses of protein expression revealed that neither BMAL1 nor PER2 had clear, anti-phase relationships in any of the cells. This is likely due to the low-resolution feature of Western blotting, where changes were too subtle to be detected. Taken together, our data support the hypothesis that circadian rhythms are increasingly disrupted with malignancy in breast cancer. To provide a better understanding and more accurate analyses of these altered oscillations, luciferase reporters and real-time luminometry should be used in the future. Furthermore, next-generation sequencing can be utilized to assess connections between cancer and circadian pathways, and may provide insights to how they affect one another. Understanding the contributions of circadian rhythms and their effects on breast cancer development will be pivotal to understanding the roles of the clock in this and other diseases.

## Figures and Tables

**Table 1:** Circadian parameters for *BMAL1* and *PER2* transcripts.

Cell Line Transcript	H16N2		21PT		21MT-1	
	*BMAL1*	*PER2*	*BMAL1*	*PER2*	*BMAL1*	*PER2*
Baseline	0.910 ± 0.066	0.485 ± 0.050	0.344 ± 0.046	0.254 ± 0.040	NA	0.876 ± 0.065
Amplitude	0.432 ± 0.066	0.309 ± 0.052	0.085 ± 0.047	0.173 ± 0.032	NA	0.208 ± 0.061
Damping Rate (1/h)	−0.015 ± 0.005	−0.011 ± 0.006	−0.050 ± 0.018	−0.028 ± 0.058	NA	−0.026 ± 0.009
Phase (h)	11.402 ± 0.412	26.952 ± 0.362	6.001 ± 1.337	31.473 ± 0.711	NA	27.033 ± 0.479
Slope (/h)	0.003 ± 0.002	0.018 ± 0.002	0.023 ± 0.0029	0.032 ± 0.002	NA	0.006 ± 0.003
Period (h)	26.546 ± 0.526	26.470 ± 0.728	35.745 ± 1.905	30.621 ± 1.238	NA	27.206 ± 0.910

Error shown indicates standard error. Because curve fitting failed to represent the variance in the data (resulting in a lack of fit), characteristics associated with *BMAL1* transcripts from 21MT-1 cells are not included (NA = not applicable).

## Data Availability

Raw data are available from the authors upon request.

## Supplementary Materials

The following are available online at https://www.mdpi.com/article/10.3390/clockssleep3040042/s1, Table S1: Estimates and confidence intervals for *BMAL1* mRNA time series from H16N2. Table S2: Estimates and confidence intervals for *PER2* mRNA time series from H16N2. Table S3: Estimates and confidence intervals for *BMAL1* mRNA time series from 21PT. Table S4: Estimates and confidence intervals for *PER2* mRNA time series from 21PT. Table S5: Estimates and confidence intervals for *PER2* mRNA time series from 21MT-1. Table S6: *p*-values from rhythmicity tests for the BMAL1 protein time series. Table S7: *p*-values from rhythmicity tests for the PER2 protein time series. Figure S1: All Western blots for BMAL1 and PER2 for H16N2 cells, 21PT cells, and 21MT-1 cells. Figure S2: Relative protein expression of BMAL1 and PER2 in the 21T series of cells, as determined by Western blot.
