# Supplementary material for: Circadian Alterations Increase with Progression in a Patient-Derived Cell Culture Model of Breast Cancer
Source: Clocks Sleep. 2021 Nov 12;3(4):598–608. doi: 10.3390/clockssleep3040042 (PMC8628750; doi:10.3390/clockssleep3040042)
Supplement: Supplementary file 1 [file clockssleep-03-00042-s001.zip › clockssleep-1337353-supplementary.pdf]

Supporting Information

Circadian Alterations Increase with Progression in a Patient-Derived Cell Culture Model of Breast Cancer

Hui-Hsien Lin,[1] Stephanie R. Taylor,[2,*] and Michelle E. Farkas[1,*]

[1]Department of Chemistry, University of Massachusetts Amherst, Amherst, MA 01003, USA

[2]Department of Computer Science, Colby College, Waterville, ME 04901, USA

Supplemental Methods

Protein Isolation

Following TRIzol treatment and removal of the RNA-containing aqueous phase as described in Materials and Methods section 4.3, any residual aqueous phase and the interface were completely removed from the sample. 0.3 mL of 100% ethanol was added to the remaining organic phase for initial homogenization. Microcentrifuge tubes were inverted several times for mixing and incubated at rt for 3 min. The samples were then centrifuged at 2000 x g for 5 min at 4 °C to pellet any residual DNA. The phenol-ethanol supernatant, which contained protein, was transferred to a new microcentrifuge tube for further protein precipitation. An excess of isopropanol was added (at minimum 2X volume of the phenol-ethanol solution) to the phenol-ethanol phase, and the samples were incubated for 10 min at rt. Following incubation, the samples were centrifuged at 12,000 x g for 10 min at 4 °C to pellet proteins. The supernatants were then removed and discarded. 500 µL of 95% ethanol was added and microcentrifuge tubes inverted to wash the protein pellets. The samples were then centrifuged at 7,600 x g for 5 min at 4 °C. The supernatants were discarded, and an additional wash with 250 µL of 95% ethanol was performed. After decanting the supernatant, the pellets were air-dried for 30 min at rt. 100 µL of the optimized lysis buffer (adapted from Kopec et al. [1]; 20 mM EDTA, 140 mM NaCl, 5% SDS, 100 mM Tris, and 1% Halt™ Protease and Phosphatase Inhibitor (Thermo Fisher Scientific)) was added to each microcentrifuge tube, which was incubated for 45 min at 50 °C while shaking at 450 rpm. After incubation, all protein pellets were completely dissolved and protein concentrations were measured using a BCA assay (Fisher Scientific). All protein solutions were stored at -20 °C for further characterization.

Western Blotting

20 µg of protein per sample was electrophoretically separated on an 8% SDS polyacrylamide gel, and transferred to a PVDF membrane (Thermo Fisher Scientific). The membrane was blocked with 5% (w/v) bovine serum albumin (BSA; Fisher Scientific) in 1X TBST (150 mM NaCl, 20 mM Tris-HCl, 0.1% Tween 20) for 2 h at rt, incubated with primary antibodies against BMAL1 (Cell Signaling), PER2, and GAPDH (Proteintech) overnight at 4 °C, washed three times with 1X TBST, and incubated with horseradish peroxidase-conjugated goat-anti-rabbit IgG secondary antibody (Thermo Fisher) for 2 h at rt. Immunoblots were imaged using an enhanced chemiluminescence reagent (ECL; Thermo Fisher) via a G:Box iChemi XT imaging system (GeneSys). Band intensities were analyzed via ImageJ. For Western Blots, N=3 biological replicates were used per cell line and time point, with a single technical replicate for each.

Table S1. Estimates and confidence intervals for *BMAL1* mRNA time-series from H16N2.

Parameter	Estimate	SE	P	CI (lb)	CI (ub)
**Baseline**	0.910271	0.065641	< 2e-16	0.79	> 1
**Amplitude**	0.431611	0.065694	6.79e-09	0.32	0.55
**Damping Rate**	-0.015291	0.004812	0.00219	-0.02	-0.007
**Phase**	11.402325	0.411827	< 2e-16	10.48	12.44
**Slope**	0.003380	0.002044	0.10254	-0.0003	0.007
**Period**	26.546171	0.525560	< 2e-16	25.09	27.71

T-series with n=6 biological replicates per time-point (from two experiments, where n=3 each) were fit to a cosine curve with an exponential damping term, a baseline offset, and a linear growth term (baseline + slope*t + amplitude*exp(-dampingRate*t)*cos(2π*(t-phase)/period)). A linear least-squares fitting method was employed to estimate the parameters, the standard error for the estimate, the p-value (indicating significance against the null hypothesis that the parameter's value is 0), and the lower and upper bounds for the 95% confidence intervals.

Table S2. Estimates and confidence intervals for *PER2* mRNA time-series from H16N2.

Parameter	Estimate	SE	P	CI (lb)	CI (ub)
**Baseline**	0.485300	0.049792	8.64e-15	0.39	0.59
**Amplitude**	0.308904	0.052479	1.15e-07	0.22	0.43
**Damping Rate**	-0.010851	0.006017	0.0755	-0.02	0.002
**Phase**	26.952060	0.362009	< 2e-16	26.26	27.72
**Slope**	0.017831	0.002140	3.68e-12	0.01	0.02
**Period**	26.470176	0.728001	< 2e-16	25.01	28.21

T-series with n=6 biological replicates per time-point (from two experiments, where n=3 each) were fit to a cosine curve with an exponential damping term, a baseline offset, and a linear growth term (baseline + slope*t + amplitude*exp(-dampingRate*t)*cos(2π*(t-phase)/period)). A linear least-squares fitting method was employed to estimate the parameters, the standard error for the estimate, the p-value (indicating significance against the null hypothesis that the parameter's value is 0), and the lower and upper bounds for the 95% confidence intervals.

Table S3. Estimates and confidence intervals for *BMAL1* mRNA time-series from 21PT.

Parameter	Estimate	SE	P	CI (lb)	CI (ub)
**Baseline**	0.343636	0.046169	1.67e-10	0.28	0.41
**Amplitude**	0.084799	0.046876	0.07462	0.07	0.11
**Damping Rate**	-0.050000	0.017523	0.00564	<-0.05	-0.04
**Phase**	6.001186	1.337328	2.68e-05	1.28	8.96
**Slope**	0.022761	0.002906	3.16e-11	0.02	0.03
**Period**	35.745030	1.904586	< 2e-16	32.11	41.77

T-series with n=6 replicates per time-point (from two experiments, where n=3 each) were fit to a cosine curve with an exponential damping term, a baseline offset, and a linear growth term (baseline + slope*t + amplitude*exp(-dampingRate*t)*cos(2π*(t-phase)/period)). A linear least-squares fitting method was employed to estimate the parameters, the standard error for the estimate, the p-value (indicating significance against the null hypothesis that the parameter's value is 0), and the lower and upper bounds for the 95% confidence intervals.

Table S4. Estimates and confidence intervals for *PER2* mRNA time-series from 21PT.

Parameter	Estimate	SE	P	CI (lb)	CI (ub)
**Baseline**	0.253857	0.039733	1.45e-08	0.18	0.34
**Amplitude**	0.172562	0.031872	7.72e-07	0.12	0.23
**Damping Rate**	-0.027785	0.005818	9.17e-06	-0.04	-0.02
**Phase**	31.473339	0.710592	< 2e-16	30.1	33.2
**Slope**	0.032126	0.002479	< 2e-16	0.03	0.04
**Period**	30.620754	1.237780	< 2e-16	28.4	34.27

T-series with n=6 biological replicates per time-point (from two experiments, where n=3 each) were fit to a cosine curve with an exponential damping term, a baseline offset, and a linear growth term (baseline + slope*t + amplitude*exp(-dampingRate*t)*cos(2π*(t-phase)/period)). A linear least-squares fitting method was employed to estimate the parameters, the standard error for the estimate, the p-value (indicating significance against the null hypothesis that the parameter's value is 0), and the lower and upper bounds for the 95% confidence intervals.

Table S5. Estimates and confidence intervals for *PER2* mRNA time-series from 21MT-1.

Parameter	Estimate	SE	P	CI (lb)	CI (ub)
**Baseline**	0.875525	0.065397	< 2e-16	0.75	> 1
**Amplitude**	0.208302	0.060829	0.00105	0.11	0.34
**Damping Rate**	-0.025844	0.009158	0.00625	-0.04	-0.008
**Phase**	27.033438	0.479048	< 2e-16	26.04	28.00
**Slope**	0.005864	0.002500	0.02194	0.0008	0.01
**Period**	27.205905	0.910427	< 2e-16	25.4	29.36

T-series with n=5 biological replicates (from two experiments, where n=2 and n=3 for each) for 4 of the time-points and 6 replicates (from two experiments, where n=3 each) for the remaining time-points were fit to a cosine curve with an exponential damping term, a baseline offset, and a linear growth term (baseline + slope*t + amplitude*exp(-dampingRate*t)*cos(2π*(t-phase)/period)). A linear least-squares fitting method was employed to estimate the parameters, the standard error for the estimate, the p-value (indicating significance against the null hypothesis that the parameter's value is 0), and the lower and upper bounds for the 95% confidence intervals.

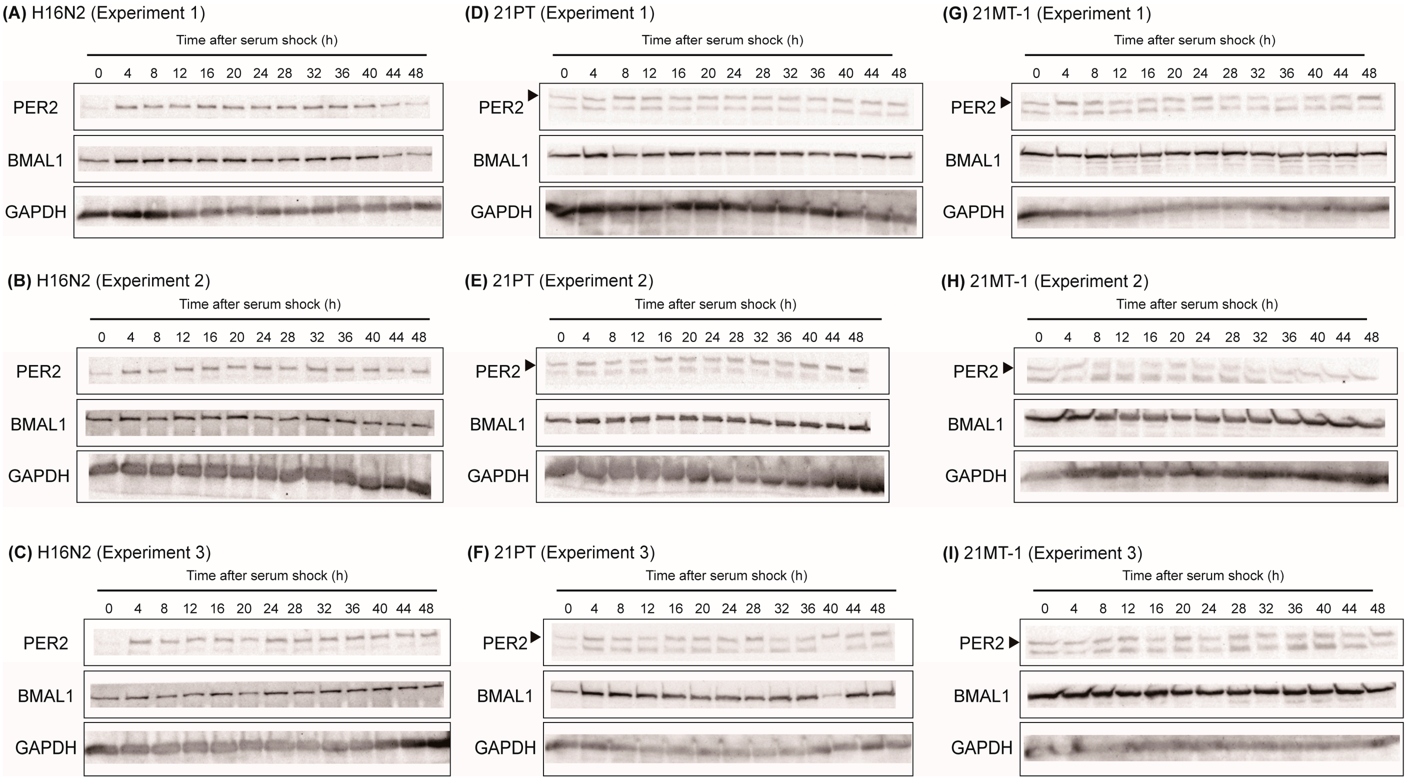

Figure S1. All western blots for BMAL1 and PER2 for **(A-C)** H16N2 cells, **(D-F)** 21PT cells, and **(G-I)** 21MT-1 cells. Each numerical experiment (1-3) denotes a separate biological replicate. A single technical replicate was performed for each.

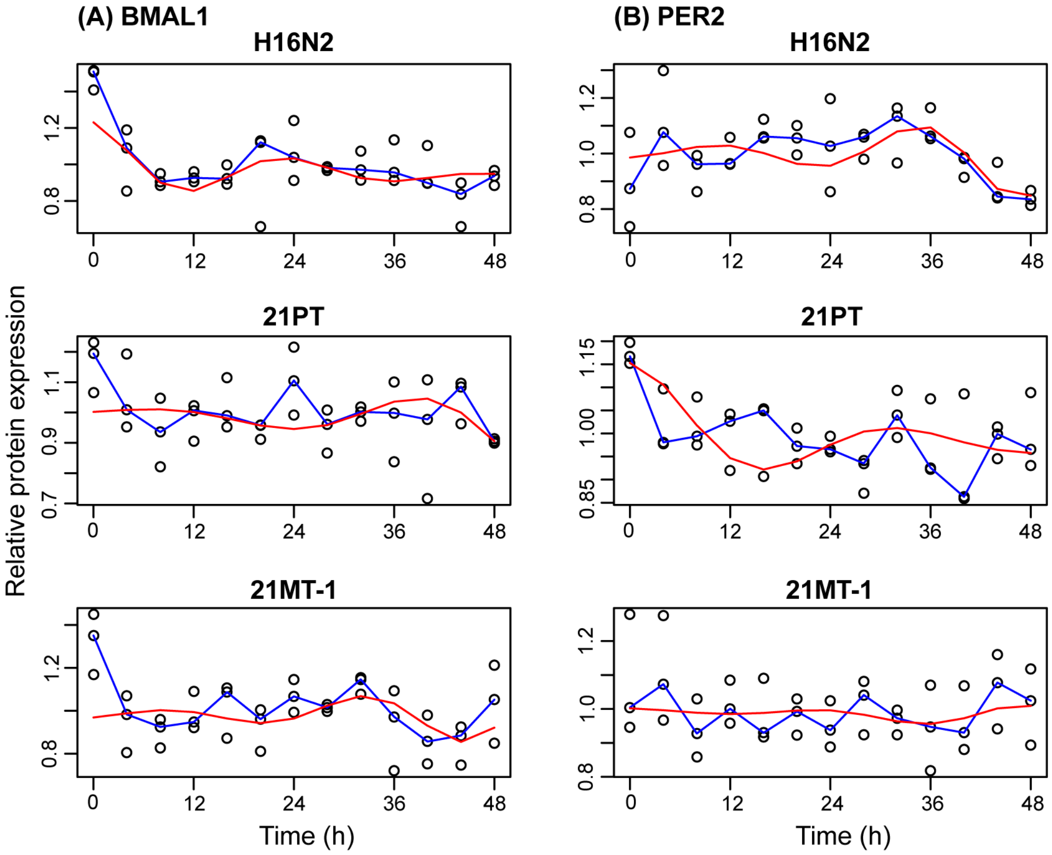

Figure S2. Relative protein expression of BMAL1 and PER2 in the 21T series of cells, as determined by western blot. Expression is shown relative to the mean over time. The median signal is shown in blue and the best-fit damped cosine curve is shown in red. The coefficient of determination (R^2) identifies low-quality fits for all time-series (R^2=0.45 for BMAL1 and 0.34 for PER2 in H16N2, -0.01 for BMAL1 and 0.21 for PER2 in 21PT, and 0.11 for BMAL1 and 0.08 for PER2 in 21MT-1).

Table S6. P-values from rhythmicity tests for the BMAL1 protein time-series.

	Rain20	Rain24	Rain28	JTK	LSR	ECHO
N	0.601	0.063	1.22e-04	2.72e-02	0.054	9.94e-06
PT	0.060	0.874	0.415	0.852	0.788	7.52e-05
MT	0.758	1.21e-02	2.40e-03	3.24e-02	3.60e-03	0.499

For each cell line, each time-series with n=3 biological replicates per time-point was tested for rhythmicity, with each of 6 tests. Rain20, Rain24, and Rain28 indicate RAIN with test periods of 20 h, 24 h, and 28h, respectively; JTK indicates JTK-Cycle; LSR indicates the Lomb-Scargle Permutation test. N=H16N2; PT=21PT; MT=21MT-1

Table S7. P-values from rhythmicity tests for the PER2 protein time-series.

	Rain20	Rain24	Rain28	JTK	LSR	ECHO
N	3.67e-02	0.139	0.176	0.379	0.099	6.59e-03
PT	0.847	0.793	0.471	1.000	0.142	0.752
MT	0.884	0.693	0.862	1.000	0.795	0.064

For each cell line, each time-series with n=3 biological replicates per time-point was tested for rhythmicity, with each of 6 tests. Rain20, Rain24, and Rain28 indicate RAIN with test periods of 20 h, 24 h, and 28h, respectively; JTK indicates JTK-Cycle; LSR indicates the Lomb-Scargle Permutation test. N=H16N2; PT=21PT; MT=21MT-1.

Reference

1. Kopec AM, Rivera PD, Lacagnina MJ, Hanamsagar R, Bilbo SD. Optimized solubilization of TRIzol-precipitated protein permits Western blotting analysis to maximize data available from brain tissue. *J Neurosci Methods.* 2017;280:64-76.
